# The association of executive functions and physical fitness with cognitive-motor multitasking in a street crossing scenario

**DOI:** 10.1038/s41598-022-26438-x

**Published:** 2023-01-13

**Authors:** Melanie Mack, Robert Stojan, Otmar Bock, Claudia Voelcker-Rehage

**Affiliations:** 1 Department of Neuromotor Behavior and Exercise, Institute of Sport and Exercise Sciences, University of Münster, Wilhelm-Schickard-Straße 8, 48149 Muenster, Germany; 2 Institute of Human Movement Science and Health, Chemnitz University of Technology, Thueringer Weg 11, 09126 Chemnitz, Germany; 3 Institute of Exercise Training and Sport Informatics, German Sport University, Am Sportpark Muengersdorf 6, 50927 Cologne, Germany

**Keywords:** Human behaviour, Risk factors

## Abstract

Age-related decline in cognitive-motor multitasking performance has been attributed to declines in executive functions and physical fitness (motor coordinative fitness and cardiovascular fitness). It has been suggested that those cognitive and physical resources strongly depend on lifestyle factors such as long-term regular physical activity and cognitive engagement. Although research suggests that there is covariation between components of executive functions and physical fitness, the interdependence between these components for cognitive-motor multitasking performance is not yet clear. The aim of the study was to examine the contribution and interrelationship between executive functions, motor coordinative fitness, and cardiovascular fitness on street crossing while multitasking. We used the more ecologically valid scenario to obtain results that might be directly transferable to daily life situation. Data from 50 healthy older adults (65–75 years, 17 females, recruited in two different cities in Germany) were analyzed. Participants’ executive functions (composite score including six tests), motor coordinative fitness (composite score including five tests), and cardiovascular fitness (spiroergometry), as well as their street crossing performance while multitasking were assessed. Street crossing was tested under single-task (crossing a two-line road), and multitask conditions (crossing a two-line road while typing numbers on a keypad as simulation of mobile phone use). Street crossing performance was assessed by use of cognitive outcomes (typing, crossing failures) and motor outcomes (stay time, crossing speed). Linear mixed-effects models showed beneficial main effects of executive functions for typing (*p* = 0.004) and crossing failures (*p* = 0.023), and a beneficial main effect of motor coordinative fitness for stay time (*p* = 0.043). Commonality analysis revealed that the proportion of variance commonly explained by executive functions, motor coordinative fitness, and cardiovascular fitness was small for all street crossing outcomes. For typing and crossing failures (cognitive outcomes), the results further showed a higher relative contribution of executive functions compared to motor coordinative fitness and cardiovascular fitness. For stay time (motor outcome), the results correspondingly revealed a higher relative contribution of motor coordinative fitness compared to executive functions and cardiovascular fitness. The findings suggest that during cognitive-motor multitasking in everyday life, task performance is determined by the components of executive functions and physical fitness related to the specific task demands. Since multitasking in everyday life includes cognitive and motor tasks, it seems to be important to maintain both executive functions and physical fitness for independent living up to old age.

## Introduction

Mobility is an essential component of an active and independent lifestyle. It includes walking and other posture-related tasks, such as stopping or standing still. Those motor tasks are often combined with additional cognitively demanding tasks, such as watching for traffic or using a mobile phone^1^. This simultaneous performance of the motor task walking with an additional cognitive task is referred to as cognitive-motor multitasking. It is well established that cognitive-motor multitasking often results in task interference, i.e., in performance losses in either the cognitive task, the motor task, or both tasks^2,3^. Resource models of attention suggest, that individuals have only a limited pool of a resources to draw on when performing tasks. When the processing of two tasks overlap, they compete for limited cognitive resources, typically leading to the aforementioned performance degradation (c.f., resource models from Kahneman^4^, and Wickens^5^).

Task interference in multitasking is more pronounced in older than in young adults^2,6,7^. Numerous and overlapping structural and functional changes in the aging brain has been attributed to those age-related performance losses as they are suggested to lead to a reduction in cognitive resources, and with that to higher compensatory trade-offs^8–11^. Age-related declines in cognitive-motor multitasking are characterized by intra- and interindividual differences^12^. For example, some individuals exhibit particularly large performance decrements in tasks with high cognitive demands, while having no performance decrements in tasks with high motor demands. In turn, for other individuals, it is the opposite. It is suggested, that individual differences in cognitive-motor performance are associated with individual differences in the age-related neurobiological adaptions. The developmental course of these neurobiological adaptions is thought to be influenced by lifestyle factors such as physical activity and intellectual engagement, which are assumed to be related to different levels of cognitive functioning (especially executive functions) and physical fitness^11,13^.

### Executive functions and cognitive-motor multitasking

Executive functions refer to higher-order cognitive control processes that supervise the processing of concurrent tasks and allocate attention^14^. They are involved in producing effective and goal directed actions^14^, and are proposed to be closely associated with cognitive-motor multitasking^6,15^. In particular, executive functions seem to be affected by the aging process because the underlying brain structures, specifically (pre-) frontal regions, are most vulnerable to aging^16^.

Several studies have already investigated the relationship between executive functions and cognitive-motor multitasking in older adults^17–22^. Most studies revealed a positive association between executive functions and multitasking performance in motor tasks^17–19,21,22^. That relationship was influenced by different task requirements, for example, the complexity of the concurrent cognitive task, showing no significant association between executive functions and motor multitasking performance for low demanding cognitive tasks as opposed to more demanding cognitive tasks^17,21^. Only one of the above studies investigated multitasking performance for both the motor and the cognitive task, revealing that a high level of executive functions benefitted both the cognitive task and the motor task under multitasking conditions^20^.

### Physical fitness and cognitive-motor multitasking

Physical fitness is a multidimensional concept that comprises various health- and skill-related attributes, including cardiovascular and motor coordinative fitness^23^. Both cardiovascular and motor coordinative fitness might benefit cognitive-motor multitasking but through different (neuro-) biological mechanisms^13,24,25^. Cardiovascular fitness refers to the physical work capacity of an individual, in the form of oxygen capacity per kilogram of body weight over time^23,26^. Motor coordinative fitness includes sensory and higher-level cognitive control processes that ensure anticipation and adaptation to act and behave meaningfully. It comprises different components such as movement speed, balance and coordination^25^. A range of cross-sectional and longitudinal studies revealed that higher cardiovascular fitness benefits motor coordinative fitness^27^, and that a higher cardiovascular and a higher motor coordinative fitness benefits cognition in older adults^25,28,29^.

#### Cardiovascular fitness

A higher level of cardiovascular fitness seems to increase cognitive resources by enhancing the brain's efficiency and reserve capacity^30–33^. Following these findings and the theoretical background of the resource models, it might be suggested that individuals with higher cardiovascular fitness may not only have a better physical constitution, but also more cognitive resources available during cognitive-motor multitasking than individuals with lower cardiovascular fitness levels. Depending on the task characteristics and the individual's cognitive functioning and physical fitness, available resources might be devoted to the motor or cognitive or both tasks, and thereby decrease cognitive-motor interference (see also "posture first" hypothesis^34^).

However, while there is comprehensive research in the aging literature on the beneficial effects of cardiovascular fitness on neurocognitive functions^35,36^, to the authors’ knowledge, only two studies examined the impact of cardiovascular fitness on cognitive-motor multitasking in older adults^37,38^. Both studies assessed multitasking performance for the motor and the cognitive task, revealing a positive association between cardiovascular fitness and multitasking performance for the motor task (i.e., walking speed). The task's difficulty influenced the beneficial effect of cardiovascular fitness on multitasking performance of the cognitive task (modified Stroop color word test). More complex tasks appear to be more strongly associated with cardiovascular fitness than less complex tasks^38^.

#### Motor coordinative fitness

Aging leads to lower functioning of the central and peripheral nervous system, as well as the neuromuscular system^39^. Those decrements have a negative impact on motor coordinative fitness, which is composed of behavioral parameters such as movement speed, temporal and spatial movement variability, or multi-joint coordination^39,40^. All these parameters play a role in postural control tasks, such as standing and walking. To compensate for decreasing motor coordinative fitness and maintaining balance and a safe and stable gait, older adults seem to rely on cognitive control processes^10^. Accordingly, a higher motor coordinative fitness would tie up fewer cognitive resources, which would then be available for additional tasks. This is also in line with findings from motor imagery studies, which suggests that the lower the motor automatization of gait, the more cognitive resources are needed for the walking task, resulting in fewer cognitive resources available for the concurrent cognitive task^41,42^.

A small line of research investigated the association between motor coordinative fitness and cognitive-motor multitasking in older adults^17–19,43,44^. Except for^43^, all studies found a positive association between motor coordinative fitness and motor multitasking performance. However, none of the latter studies assessed the performance of the concurrent cognitive task, leaving open whether motor coordinative fitness benefits only the multitasking performance of the motor task or the cognitive task. For example, the “posture first” hypothesis suggests that older adults prioritize gait over cognitive performance when walking under multitasking conditions^34,45^. As a result, it could be assumed that participants with a lower level of motor coordinative fitness would need additional cognitive resources to perform the motor task, leading to lower cognitive performance under multitasking conditions. On the other hand, participants with a higher level of coordinative motor fitness would have cognitive resources left for the cognitive tasks. Accordingly, a positive association between motor coordinative fitness and cognitive multitasking performance would occur.

### Interplay of physical fitness and executive functions

Research suggest that there is covariation between executive functions and physical fitness^28,46,47^, which is also reflected in their interrelated effects on multitasking performance^13^. For multitask driving, it could be demonstrated that there are direct and indirect effects of motor coordinative and cardiovascular fitness on multitasking performance. Both physical fitness components were related to executive functions, directly affecting multitasking driving. Those effects thereby varies for different driving outcomes^13^. Since walking differ from driving in terms of motor and cognitive requirements, results might not be transferable to multitask walking.

### Rationale

As we detailed in the preceding paragraphs, several studies evaluated—mostly within laboratory paradigms—the association between single components of executive functions and physical fitness with single outcomes of cognitive-motor multitasking performance in older adults. Although the results of those studies confirm the assumptions of the beneficial effects of executive functions and physical fitness on multitasking, some questions remain unanswered:

1. What is the relative and interrelating role of the different components of executive functions and physical fitness (motor coordinative fitness and cardiovascular fitness) for cognitive-motor multitasking?
2. Do the effects of executive functions, motor coordinative fitness, and cardiovascular fitness differ for the various cognitive-motor multitasking outcomes?

We aimed to investigate those research questions in a more ecologically valid setting to understand the functional relevance of different components of executive functions and physical fitness for multitasking in everyday life. Many real-life situations, such as street crossing, include more complex behavioral sequences with differing cognitive and motor demands. Laboratory paradigms on the other hand present only a limited number of well-defined stimuli (e.g., differently colored shapes displayed against a featureless background), call for a single response type (e.g., a button press), and are not embedded in a natural context (i.e., they are not part of a behavioral sequence that is ultimately intended to achieve a desirable goal). Hence, laboratory paradigms differ from many real-life situations^48–51^ and the outcomes of typical laboratory experiments (e.g. cognitive outcomes such as reaction time and accuracy, or motor outcomes such as walking speed) may not be transferable to everyday-life^48,52,53^. Understanding better which components are relevant to multitasking in activities of daily living and how they are interrelated would provide insights into which components we need to pay special attention to in order to avoid age-related mobility limitations and promote successful and independent aging. This knowledge could then be used, for example, in the design of training interventions to improve cognitive-motor multitasking in older adults.

In summary, the aim of the present study was to examine the differential effects and the interrelating role of executive functions, motor coordinative fitness, and cardiovascular fitness on different cognitive-motor multitasking outcomes in a more ecologically valid cognitive-motor multitasking scenario, i.e., street crossing. This scenario was implemented in virtual reality, as already described in Janouch et al.^54^, and similar as in other studies on street crossing^55–57^. We expected that a higher multitasking performance would be associated with a higher level of executive functions, and/or motor coordinative fitness, and/or cardiovascular fitness, depending on the cognitive and/or motor demands of the street crossing outcomes (hypothesis I). We further expected that the relative contribution of the different components of cognitive functioning and physical fitness would vary for the different street crossing outcomes (hypothesis II).

## Results

### Linear mixed-effect model analysis

**Figure 1:**
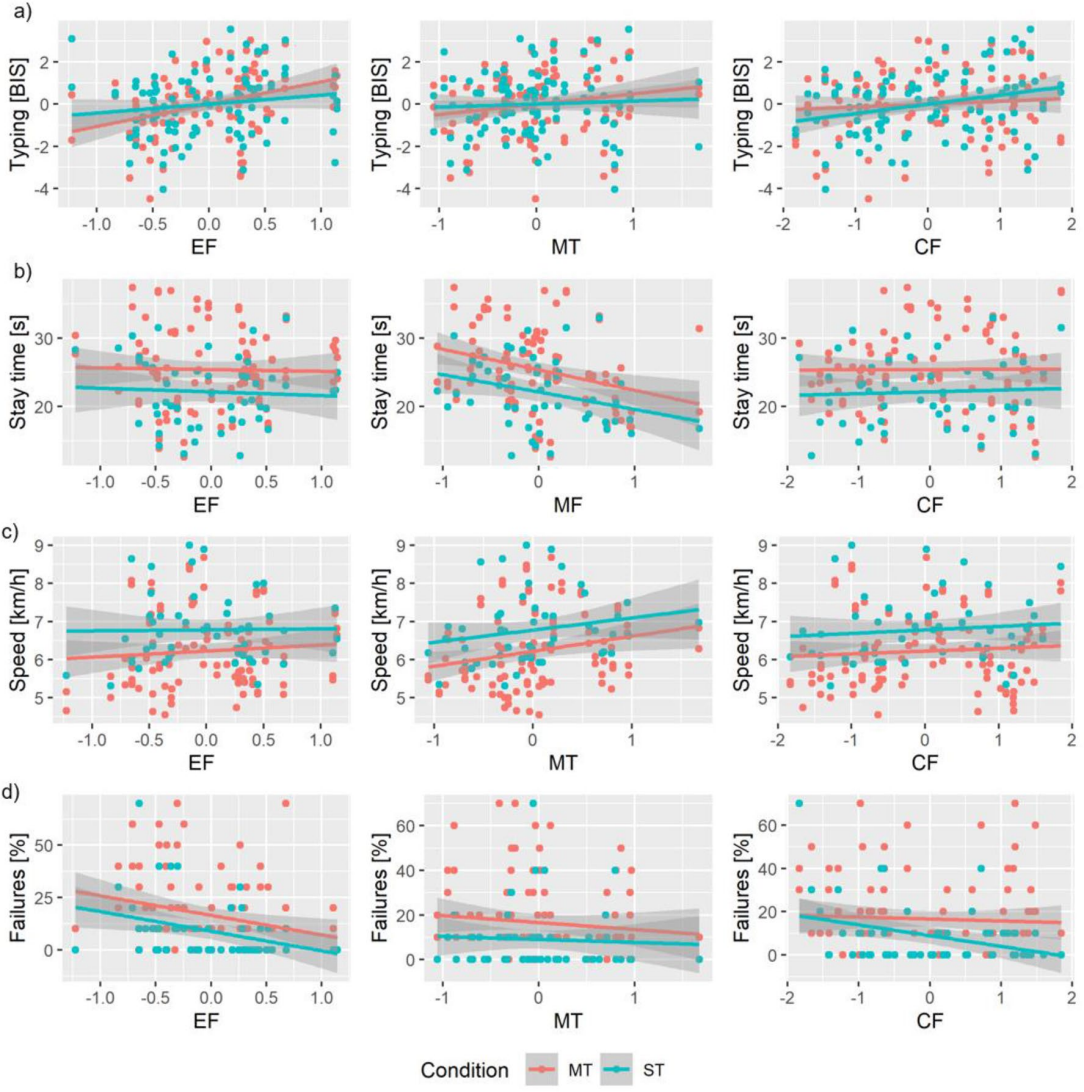
Linear mixed-effects model results showing effects of executive functions, motor coordinative fitness, and cardiovascular fitness on the outcome variables (**a**) typing, (**b**) crossing failures, (**c**) stay time, and (**d**) crossing speed, separately for single- and multitask condition. Model-predicted means and 95% CI are shown. EF = executive functions, MF = motor coordinative fitness, CF = cardiovascular fitness, ST = single-task condition, MT = multitask condition.

We performed linear mixed-effects model analysis to examine hypothesis I. Results are presented in Fig. 1, Tables 1, and 2. Non-significant results are not reported in the text below.

**Table 1:** Results of the linear mixed-effects models for typing, and crossing failures.

Predictors	Typing [BIS]			Crossing failures [%]		
	*Est*	*CI*	*p*	*Est*	*CI*	*p*
(Intercept)	0.24	− 7.90 to 8.37	0.953	− 8.92	− 100.88 to 83.04	0.846
Gender [f]	− 0.35	− 1.28 to 0.58	0.453	0.16	− 1.11 to 1.43	0.801
Age	0.00	− 0.11 to 0.12	0.935	1.92	− 8.58 to 12.41	0.715
Education	− 0.03	− 0.12 to 0.07	0.571	0.87	− 0.23 to 1.97	0.119
Cond [ST]	− 0.00	− 0.36 to 0.35	0.978	**− 7.60**	− **12.05** to **− 3.14**	**0.001**
EF	**1.08**	**0.36 to 1.80**	**0.004**	**− 10.46**	− **19.40** to **− 1.51**	**0.023**
MF	0.50	− 0.17 to 1.17	0.139	− 2.61	− 10.93 to 5.71	0.531
CF	− 0.04	− 0.51 to 0.44	0.872	0.16	− 5.55 to 5.87	0.956
Cond [ST] * EF	**− 0.70**	**− 1.39 to − 0.02**	**0.044**	0.71	− 7.84 to 9.26	0.868
Cond [ST] * MF	− 0.40	− 1.03 to 0.23	0.205	2.32	− 5.55 to 10.18	0.559
Cond [ST] * CF	***0.35***	***− 0.01 to 0.71***	***0.058***	− ***4.18***	− ***8.71 to 0.35***	***0.070***
Marginal R^2^	0.128			.286		

*Est* estimate, *CI* confidence interval, *Cond* condition, *EF* executive functions, *MF* motor coordinative fitness, *CF* cardiovascular fitness, *ST* single-task, *MT* multitask.

Significant effects (*alpha* < 0.05) are written in bold and marginal significant effects (*alpha* ≥ 0.05 and < 0.10) are written in bold and italic.

**Table 2:** Results of the linear mixed-effects models for stay time, and crossing speed.

Predictors	Stay time [s]			Crossing speed [km/h]		
	*Est*	*CI*	*p*	*Est*	*CI*	*p*
(Intercept)	17.11	− 18.24 to 52.46	0.334	9.61	2.99 to 16.22	0.005
Gender [f]	2.36	− 1.67 to 6.40	0.244	− 0.64	− 1.39 to 0.12	0.096
Age	0.13	− 0.36 to 0.62	0.593	− 0.05	− 0.14 to 0.04	0.300
Education	− 0.10	− 0.53 to 0.32	0.621	0.01	− 0.07 to 0.09	0.804
Cond [ST]	**− 3.24**	**− 4.29 to − 2.20**	**< 0.001**	**0.56**	**0.44 to 0.68**	**< 0.001**
EF	− 0.17	− 3.25 to 2.91	0.914	0.14	− 0.39 to 0.67	0.600
MF	**− 2.97**	**− 5.84 to − 0.10**	**0.043**	0.35	− 0.14 to 0.85	0.159
CF	0.94	− 1.10 to 2.98	0.360	− 0.15	− 0.52 to 0.21	0.397
Cond [ST] * EF	− 0.32	− 2.32 to 1.68	0.748	− 0.14	− 0.37 to 0.09	0.229
Cond [ST] * MF	0.42	− 1.43 to 2.26	0.651	− 0.08	− 0.29 to 0.13	0.455
Cond [ST] * CF	0.23	− 0.83 to 1.29	0.666	0.03	− 0.10 to 0.15	0.668
Marginal R^2^	0.166			0.619		

*Est* estimate, *CI* confidence interval, *Cond* condition, *EF* executive functions, *MF* motor coordinative fitness, *CF* cardiovascular fitness, *ST* single-task, *MT* multitask.

Significant effects (*alpha* < 0.05) are written in bold and marginal significant effects (*alpha* ≥ 0.05 and < 0.10) are written in bold and italic.

#### Typing

The linear mixed-effects model revealed a significant main effect for executive functions, a significant interaction effect between condition and executive functions, and a marginally significant interaction effect between condition and cardiovascular fitness. A higher level of executive functions was associated with a better typing performance, more so during multi- than single-tasking (Fig. 1a). While the main effect for cardiovascular fitness was not significant, the interaction effect of condition and cardiovascular fitness was marginally significant. Cardiovascular fitness was more strongly positively associated with typing during single- than during multitasking (Fig. 1a). Separate linear mixed-effects models for single- and multitask condition showed that during single-tasking, there was neither a main effect for executive functions (*ß* = 0.42, *p* = 0.149), nor a main effect for cardiovascular fitness (*ß* = 0.36, *p* = 0.076). During multitasking, there was a main effect for executive functions (*ß* = 1.03, *p* = 0.001), but no main effect of cardiovascular fitness on typing performance (*ß* = − 0.10, *p* = 0.636).

#### Crossing failures

The linear mixed-effects model revealed a significant main effect for condition and executive functions, and a marginally significant interaction effect between condition and cardiovascular fitness. Participants made fewer crossing failures during single- (*M* = 9.00, *SD* = 14.32) compared to multitasking (*M* = 16.6, *SD* = 18.70). A higher level of executive functions was associated with lower crossing failures for both single- and multitasking (Fig. 1b). While the main effect of cardiovascular fitness was insignificant, the interaction effect of condition by cardiovascular fitness was marginal. Cardiovascular fitness was more strongly associated with crossing failures during single- compared to multitasking. Separate linear mixed-effects models for single- and multitask conditions as post hoc tests could show that during single-tasking, there was a marginally significant effect for cardiovascular fitness (*ß* = − 4.76, *p* = 0.066) on crossing failures. During multitasking, the effect was not significant.

#### Stay time

The linear mixed-effects model revealed a significant main effect for condition and motor coordinative fitness. Participants stayed longer at the curb during multi- (*M* = 25.38 s, *SD* = 5.75 s) compared to single-tasking (*M* = 22.14 s, *SD* = 4.99 s). A higher motor coordinative fitness was associated with a lower stay time, during both single- and multitasking (Fig. 1c).

#### Crossing speed

The linear mixed-effects model revealed a significant main effect for condition and a marginally significant main effect of motor coordinative fitness. Participants crossed the road faster during single- (*M* = 6.77 km/h, *SD* = 0.91 km/h) compared to multitasking (*M* = 6.22 km/h, *SD* = 0.98 km/h). A higher motor coordinative fitness was associated with a faster crossing speed during single- and multitasking (Fig. 1d).

Summing up, there were main effects of condition for stay time, crossing speed and crossing failures with lower performance during multi- compared to single-tasking. Furthermore, we found that executive functions, motor coordinative fitness, and cardiovascular fitness effect parameters of cognitive-motor multitasking differently. The analyses showed beneficial effects of executive functions on the cognitive outcomes typing and crossing failures, as well as beneficial effects of motor coordinative fitness on the motor outcome stay time. We further found interaction effects between condition and executive functions for the cognitive outcomes typing and crossing failures. The beneficial effects were higher during multi- than single-tasking. Additionally, for those two cognitive outcomes, there was a marginally significant interaction effect between condition and cardiovascular fitness, but with lower beneficial effects during multi- compared to single-tasking.

### Commonality analysis

**Figure 2:**
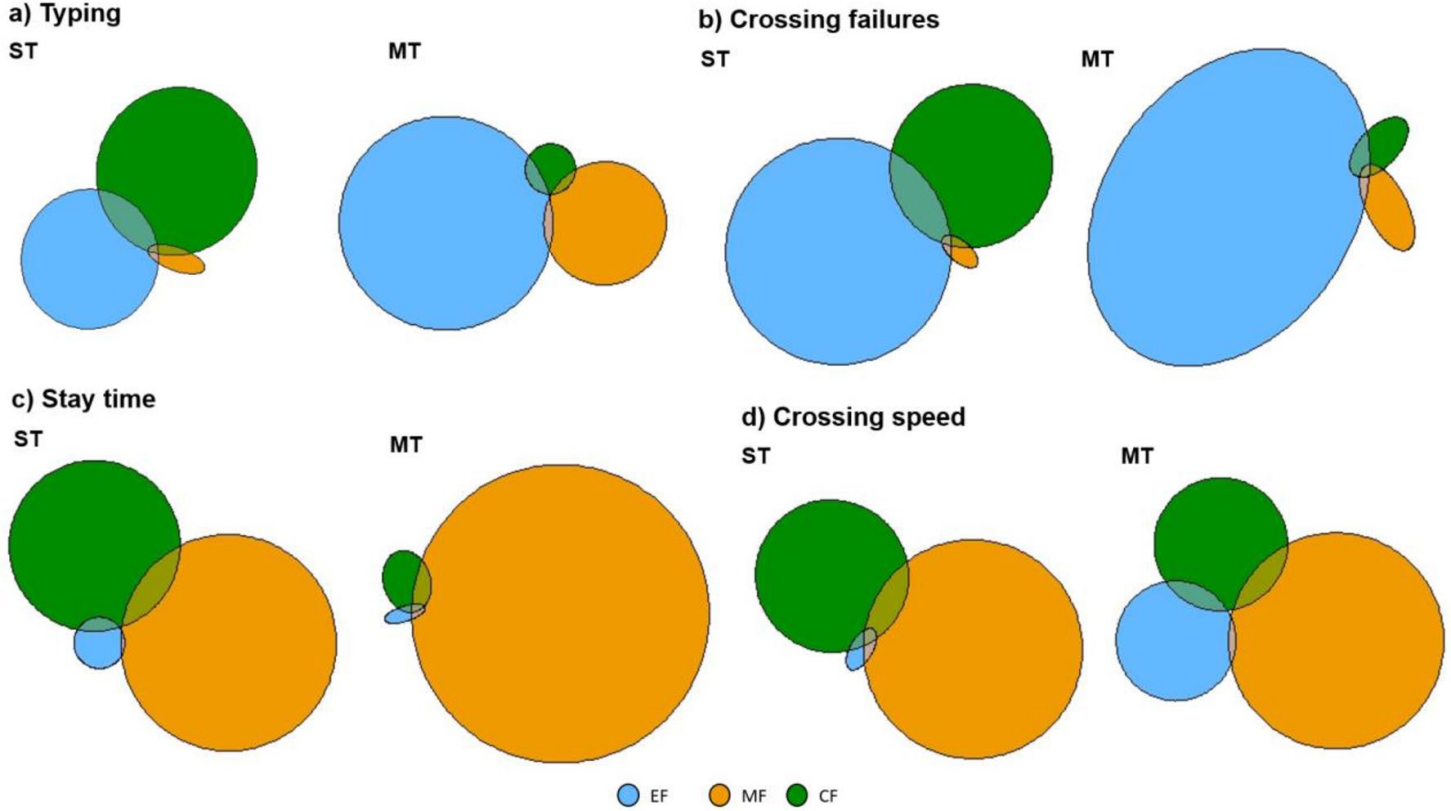
Graphical representation of the unique and shared variance of executive functions, motor coordinative fitness, and cardiovascular fitness in explaining (**a**) typing, (**b**) crossing failures, (**c**) stay time, and (**d**) crossing speed during single- and multitasking. EF = executive functions, MF = motor coordinative fitness, CF = cardiovascular fitness, MT = multitask condition, ST = single-task condition.

We performed commonality analysis to examine hypothesis II^58,59^. Results are presented in Fig. 2 and Table 3.

**Table 3:** Results of the commonality analysis for typing, crossing failures, stay time, and crossing speed.

Predictors	Typing [BIS]		Crossing failures [#]		Stay time [s]		Crossing speed [km/h]	
	Coeff	% Exp	Coeff	% Exp	Coeff	% Exp	Coeff	% Exp
**ST condition**								
Unique to EF	0.0203	17.78	0.1165	47.06	0.0025	1.55	0.0001	0.05
Unique to MF	0.0007	0.63	0.0007	0.26	0.0758	47.51	0.0293	20.49
Unique to CF	0.0306	26.77	0.0505	20.40	0.0412	25.87	0.0124	8.67
Common EF & MF	0.0001	0.05	0.0001	0.06	0.0002	0.14	0.0000	− 0.02
Common to EF & CF	0.0031	2.67	0.0094	3.81	− 0.0010	− 0.64	0.0002	0.11
Common to MF & CF	0.0004	0.39	0.0006	0.23	− 0.0045	− 2.81	− 0.0015	− 1.06
Common to EF, MF & CF	0.0000	0.04	0.0001	0.05	0.0000	0.00	0.0000	− 0.02
Total	0.1143	100	0.2576	100	0.1595	100	0.1428	100
**MT condition**								
Unique to EF	0.1092	63.74	0.0897	69.47	0.0003	0.27	0.0089	7.57
Unique to MF	0.0333	19.50	0.0031	2.44	0.0894	94.00	0.0334	28.40
Unique to CF	0.0020	1.17	0.0011	0.83	0.0013	1.37	0.0092	7.78
Common to EF & MF	0.0010	0.57	0.0003	0.21	0.0001	0.09	0.0003	0.24
Common to EF & CF	− 0.0013	− 0.78	− 0.0008	− 0.64	− 0.0001	− 0.06	− 0.0010	− 0.84
Common to MF & CF	− 0.0006	− 0.36	− 0.0001	− 0.11	− 0.0007	− 0.78	− 0.0014	− 1.18
Common to EF, MF & CF	0.0003	0.16	0.0001	0.06	0.0000	0.01	0.0001	0.05
Total	0.1713	100	0.1292	100	0.0951	100	0.1177	100

*EF* executive functions, *MF* motor coordinative fitness, *CF* cardiovascular fitness, *Coeff* commonality coefficients, *% Expl* proportion of explained variance in %.

For typing and crossing failures, during single-tasking, most of the unique variance was explained by executive functions (typing: 17.78%, crossing failures: 47.06%) and cardiovascular fitness (typing: 26.77%, crossing failures: 20.40%). Motor coordinative fitness explained only little unique variance (typing: 0.63%, crossing failures: 0.26%). The distribution of unique variance changed during single- compared to multitasking. During multitasking, the proportion of unique variance explained by executive functions (typing: 63.74%, crossing failures: 69.47%) and motor coordinative fitness (typing: 19.50%, crossing failures: 2.44%) was higher compared to single-tasking. In contrast, the proportion of unique variance explained by cardiovascular fitness decreased considerably during multitasking (typing: 1.17%, crossing failures: 0.83%).

For stay time and crossing speed, most of the unique variance during single-tasking was explained by motor coordinative fitness (stay time: 47.51%, crossing speed: 20.49%) and cardiovascular fitness (stay time: 25.87%, crossing speed: 8.67%). Executive functions explained only little unique variance (stay time: 1.55%, crossing speed: 0.05%). The distribution of unique variance changed during single- compared to multitasking. The proportion of unique explained variance by motor coordinative fitness (stay time: 94.00%, crossing speed: 28.40%) was higher during multi- compared to single-tasking. In contrast, the proportion of unique explained variance by cardiovascular fitness (stay time: 1.37%, crossing speed: 7.78%) was lower during multi- compared to single-tasking. The proportion of unique explained variance by executive functions decreased during multitasking for stay time (0.27%) and increased for crossing speed (7.57%).

The proportion of common/shared explained variance by executive functions, motor coordinative fitness, and cardiovascular fitness (combination of two or three variables) was only small under both single- and multitasking across all four dependent variables (− 2.81 to 3.81%) compared with the in part large values of unique explained variance. The obtained values were thereby partly negative and partly positive for all dependent variables. Negative values can occur in case of suppression or when some of the correlations between the predictor variables (e.g. executive functions and motor coordinative fitness) have opposite signs (for more detailed explanations see^60,61^). Because the values are rather small in contrast to values of the unique variance, we have not interpreted them here.

## Discussion

In accordance with our hypothesis, the findings demonstrated a beneficial main effect of executive functions, as well as a higher relative contribution of executive functions compared to motor coordinative fitness and cardiovascular fitness on typing and crossing failures (cognitive outcomes) while street crossing. For stay time (motor outcome), the results showed correspondingly a beneficial main effect of motor coordinative fitness, as well as higher relative contribution of motor coordinative fitness compared to executive functions and cardiovascular fitness. With that we are the first study that obtained results on the relative and interrelating role of executive function, motor coordinative fitness, and cardiovascular fitness for more ecologically valid actions involving more complex tasks.

Several studies on multitask-walking focused on isolated components of executive functions^17–22^ or physical fitness (motor coordinative fitness^17–19,43,44^, or cardiovascular fitness^37,38^). These studies revealed a positive association between those individual components and cognitive-motor multitasking (either cognitive or motor outcomes). Our study builds on this prior work by examining them jointly and was thus able to show that the relationship is not universal. Similar to what has already been shown for multitasking while driving^13^, the association between cognitive-motor multitasking performance while street crossing and different components of executive functions and physical fitness may depend on the requirements of the single subtasks while street crossing (i.e., different street crossing outcomes). Typing and crossing a road without getting hit by a car might particularly involve higher-order cognitive functions, such as action planning (e.g., planning the road crossing), task switching (e.g., switching between tracking the traffic and walking), or working memory (e.g., keeping the 3-digit numbers in mind). Stay time at the curb and crossing speed require participants abilities to start walking when the street is clear, to stop quickly if a car arrives unexpectedly, and to adapt the walking pace to the perceived available time—all aspects strongly related to motor coordinative fitness^62^.

Commonality analysis demonstrated, that for the outcome stay time, the amount of unique explained variance by motor coordinative fitness was higher during multi- than during single-tasking. The results of the cognitive outcomes typing, and crossing failures showed the same pattern for executive functions. Additionally, linear mixed-effects model analysis revealed a significant interaction effect between condition (single-tasking vs. multitasking), and executive functions for typing, indicating that the beneficial effect of executive functions on performance was higher during multi- compared to single-tasking. These results suggest that when individuals multitask, their level of executive functions and physical fitness might serve as “preconditions” to appropriately meet the demands of the specific tasks. This seems to be more pronounced than when engaging in a single task. It might be assumed that the cognitive/motor demands increase by adding additional tasks, causing cognitive/motor performance to be more strongly dependent on individual’s level of executive functions/motor coordinative fitness.

Findings for cardiovascular fitness differ from those for executive function, and motor coordinative fitness in two aspects: First, the proportion of unique variance is quite similar across the different cognitive and motor street crossing outcomes. Second, the proportion does not increase from single- to multitasking but decreases. None of the tasks used in our street crossing scenario place high metabolic demands. Thus, if we assume that multitasking performance while street crossing is determined by the components of executive functions and physical fitness related to the demands of the specific tasks, it seems only logical that cardiovascular fitness determines performance of the different cognitive and motor street crossing outcomes in a similar way. The assumption that cognitive/motor performance is even more strongly dependent on individual’s level of executive functions/motor coordinative fitness in multitask compared to single-task condition could further explain why the influence of cardiovascular fitness decreases from single- to multitasking.

### Strength and limitations

We provide additional value to previously conducted studies by conducting our experiment within a more ecologically valid scenario^52^, and by addressing the interrelationship between the different components executive functions, motor coordinative fitness, and cardiovascular fitness during cognitive-motor multitasking. However, studies with more ecologically valid scenarios have their limitations. First, the results might be influenced by confounding variables such as task irrelevant visual or auditory information. The influence of these variables could be investigated by a direct comparison of the same task between ecologically more valid and laboratory paradigms. Moreover, ecologically more valid experiments still differ from the real world, which could influence task performance. On the one hand, they might have a game character that shifts task priorities. Performing the typing task correctly might be suddenly more important than not getting hit by a car. On the other hand, subjects who walk confidently on the floor may feel unstable on the treadmill, which could change their gait behavior. Finally, with our study, we cannot make causal inferences between executive functions and physical fitness with cognitive-motor multitasking. Thus, future work is needed to investigate the effects of cognitive and physical training interventions on the interrelating role of those different components for multitasking in older adults.

### Conclusion and outlook

Crossing the street is an attention-demanding task. Any additional concurrent task, such as talking to someone, mentally going through a shopping list or writing messages on a smartphone, might disrupt street crossing performance and, in the worst case, lead to accidents. Street crossing while typing is further a multitask including complex behavioral sequences (c.f., outcomes) with differential cognitive and motor demands. The performance of the individual outcomes (typing, crossing failures, stay time, and crossing speed) taken together gives the total multitasking performance. Thus, each outcome contributes its part to whether or not for example an accident occurs. Given, the performance of different outcomes is determined by the components of executive functions and physical fitness related to the specific demands of those outcomes, it could be concluded that for successful multitasking in everyday life of older adults, it seems to be important to maintain both a high level of executive functions, and a high level of motor coordinative fitness.

Research on humans, but also on animals has already pointed out that different types of training (cognitive training, motor coordinative training, or cardiovascular training) might affect structure and function of brain and body, and therewith improve performance of different cognitive and motors tasks differentially^24,63,64^. From a practical point of view, the results of those intervention studies in combination with our study results call for multicomponent training regimes (e.g., combining cognitive, motor, and cardiovascular training) to comprehensively train cognitive-motor multitasking. Moreover, as the slope and pace of the aging process differs between individuals^12^, and also within individuals for different components of executive functions and physical fitness^44^, highly individualized training regimes are required. Individuals with low executive functions might, for example, benefit especially from cognitive training and individuals with low motor coordinative fitness might especially benefit from motor training. Further research should therefore investigate the effectiveness of different training regimes, and especially tailored training for cognitive-motor multitasking. All following studies examining the influence of different components of cognitive functioning and physical fitness on cognitive-motor multitasking in both laboratory and more ecologically valid settings should be aware that the demands of the chosen tasks are likely to influence the results.

## Methods

### Study design and participants

This study was part of a larger project within the German Research Foundation Priority Program SPP 1772 “Multitasking”. It was conducted at the German Sport University Cologne, and at the Chemnitz University of Technology, Germany, and approved by the ethics committee. All participants provided written informed consent before participating in this study, which was designed in accordance with ethical principles based on the Declaration of Helsinki.

The participants underwent a comprehensive test battery to assess their cognitive, and physical fitness, and their cognitive-motor multitasking performance. The latter was assessed in two everyday-like virtual scenarios: car driving and street crossing. Findings regarding the structure of executive functions in young and older adults^65^, age-related cognitive-motor interference in driving^51^, and street crossing performance^54^ have already been published elsewhere.

Older adults (*N* = 61) between 65 and 75 years of age took part in this study, from which data of 50 participants were analyzed (reasons for exclusion are described below in the respective paragraphs). Demographic characteristics are presented in Table 4. Participants were recruited via homepage announcements, local senior networks, newspaper articles, and postings at public places and social media. Participants were allowed to participate in the study if they met the following self-reported criteria: (1) aged between 65 and 75 years, (2) BMI < 30, (3) good physical and mental health (absence of physical, neurocognitive, or psychological medical disorders), (4) no red-green color blindness, (5) regular driving activities of at least one trip per week during the last six months, (6) being able to walk for at least 30 min without any assistance. Besides, all participants had to obtain a physician’s health clearance (exercise electrocardiogram, ECG) within the last six months. All self-reported criteria were requested during a telephone interview. Subsequent screening assessed: (1) overall cognition by the Mini-Mental State Examination (MMSE^66^) with a cutoff score of 27/30, (2) visual acuity by the Freiburg vision test (FrACT 3.9.0^67^) with a cutoff score of 20/60, (3) language comprehension by the Freiburg speech intelligibility test^68^ with a cutoff score of 50% word recognition at the best hearing level, and (4) handedness by the Edinburgh Handedness Inventory^69^. No person had to be excluded because of their screening outcomes. Five participants were left-handed, one was ambidextrous but used the right hand for typing, and all others were right-handed. Participants who regularly wore vision or hearing aids kept doing so during testing. The study protocol was approved by the Ethics Committees of the German Sports University Cologne (Nr.: 27/2015).

**Table 4:** Participants’ demographic information.

	Included (*n* = 50) *M* (*SD*) or *n*	Excluded (*n* = 11) *M* (*SD*) or *n*	Difference *p* (*t* or *X*^*2*^)
Age (years)	69.99 (2.97)	70.36 (3.01)	0.636 (0.48)^t^
Sex (male / female)	33/17	6/5	0.711 (0.14)^x^
BMI (kg/m^2^)	25.31 (2.93)	24.91 (2.11)	0.985 (0.02)^t^
MMSE (0–30)	29.16 (0.87)	29.09 (0.83)	0.807 (− 0.25)^t^
Education (years)	15.62 (3.22)	15.27 (1.85)	0.569 (− 0.58)^x^

Means (*M*) and standard deviations (*SD*) or number of cases (*n*) are presented.

*BMI* body mass index, *MMSE* mini mental state examination, *t* two sample t-test, *x* Chi-square test.

### Cognitive-motor multitasking test

#### Hard- and software

**Figure 3:**
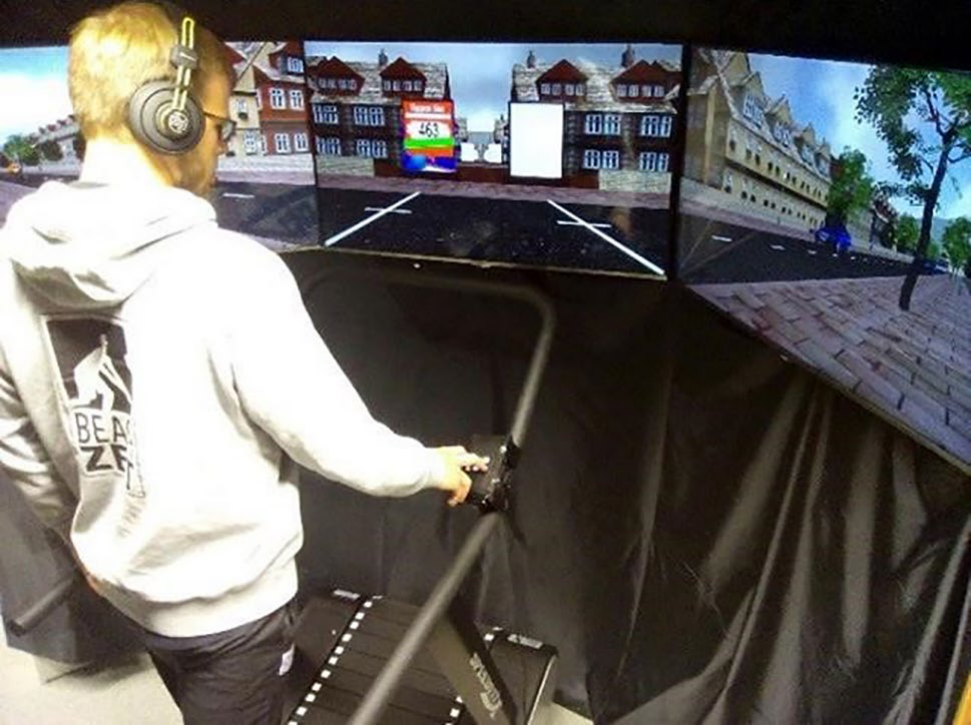
Street crossing simulator set up. Photograph of a participant walking on the treadmill through a virtual back alley towards a street. The participant wears headphone through which auditory stimuli are presented. The keyboard on which the manual responses are given is installed on the right side of the treadmill. Figure reprinted from "Cognitive-motor interference in an ecologically valid street crossing scenario", by Janouch, C. et al., 2018, Frontiers in Psychology, 9, Article 602. CC BY 4.0.

The street crossing simulator consisted of a non-motorized treadmill (DRAX, Speedfit 1000c, Vibrafit, Solms) and three 46" TV flat screens that featured a 195-degree horizontal field of view. A virtual scenario, depicting a virtual street from the first-person perspective, was projected onto the screens (Fig. 3). It consisted of city buildings grouped around a back alley and a tree-lined street with two lanes. The scenario was designed with a modified version of the commercially available driving simulator software (Carnetsoft BV, Version 8.9, Groningen, The Netherlands^70^). Treadmill speed was registered optoelectronically and synchronized with the virtual scenario, allowing participants to progress forward through the virtual scenario by walking forward on the treadmill. Participants were secured by a drop guard, and were able to keep their non-dominant hand on a laterally attached handrail for the entire test duration. A microphone registered verbal responses. A keypad with numbers from 1 to 6, attached within easy reach of the participants’ preferred hand, registered typing responses.

#### Tasks

Participants performed the following tasks under single-task condition and under multitask condition: street crossing and two cognitively demanding tasks. The cognitively demanding tasks mimicked activities that are often performed in everyday life while crossing a street, namely using a smartphone and rehearsing a shopping list. Since the cognitively demanding tasks were presented either in a visual or an auditory mode, participants performed overall five single-task blocks and four multitask blocks.

##### *Single-task street crossing* (STcross)

At the beginning of this task, participants stood still on the treadmill while the virtual scenario was displayed. On the street, cars were passing from left to right in the lane closer to the participants, and from right to left in the lane farther from the participants. All cars moved at a constant speed of 50 km/h. Inter-vehicle gaps increased with each car according to the recurrent sequence 2, 2.5, 3, 3.5, 4, 4.5, 5, 5.5, 6 s. Cars on the distal lane reached the display center one second later than the cars on the proximal lane. The participants' task was to walk through the side alley towards the street, to check for a suitable gap between the oncoming vehicles, and to cross the street without getting hit by a car. They were asked to walk at the pace at which they would cross a street in real life. One trial was completed when participants reached the opposite walkway, when they were hit by a car, or after 80 s elapsed.

##### Single-task typing

These tasks mimicked smartphone usage and were presented visually and auditorily. Participants stood still on the treadmill. To keep visual stimulation comparable to that in the other tasks, the virtual reality display presented the same sequence of events as during STcross (approach through the side alley towards the street, wait for a suitable gap in traffic, and street crossing). Presentation time was similar to that in STcross. Three-digit numbers with digits between 1 and 6 were presented sequentially, with an inter-stimulus interval of about 4 s. Each number was presented either for 4 s visually on billboards across the street (STtype_vis), or auditorily for about 1.7 s through headphones (STtype_aud). Stimuli were presented repeatedly in a random order from trial onset to trial end (when the visual display reached the opposite walkway). Participants were asked to depress the presented three-digit numbers on the keypad with their dominant hand as quickly and accurately as possible.

##### Single-task shopping

These tasks mimicked the rehearsal of a shopping list and were presented visually and auditorily. Grocery products were sequentially presented either visually on a billboard across the street (STshop_vis) or auditorily through headphones (STshop_aud). Participants were asked to indicate products that were displayed twice with a clearly spoken “yes”. To limit the degree of complexity of the present communication and because earlier analyses revealed that the typing tasks exhibited stronger task interference than the shopping tasks^54^, we analyzed only the typing task. Nevertheless, since the task is necessary to explain the design, we mention it briefly in this section. However, it is important to note that the typing task was not administered alone but intermixed with the shopping task to mimic the diversity of everyday multitasking. The mix of tasks probably brought about switch costs, as discussed in a car driving simulator study^51^.

The four multitask blocks (MTtype_vis, MTtype_aud, MTshop_vis and MTshop_aud) were implemented by combining the street crossing task with each typing task and each shopping task. The tasks were performed as described above but concurrently. Participants were instructed not to prioritize either task and respond as accurately and quickly as possible. Seven to ten typing or shopping stimuli were presented, depending on the participants’ crossing time on a given trial.

#### Design

The test was arranged in a Latin square design. Participants walked actively during 50 trials on the treadmill. On those trials, task blocks (STcross, MTtype_vis, MTtype_aud, MTshop_vis and MTshop_aud) were presented ten times each in a mixed sequence (active trials). On 40 further trials, participants stood still on the treadmill while the task blocks STtype_aud, STtype_vis, STshop_aud, and STshop_vis were presented ten times each in a mixed sequence (passive trials). To minimize physical fatigue resulting from long periods of walking, blocks of ten active trials were alternated with blocks of ten passive trials, starting with an active block.

#### Outcomes

We calculated four different outcomes with different cognitive and motor demands from the data, which were assumed to be relevant parameters to assess street crossing performance during multitasking.

##### Typing

First, typing accuracy (in %) was calculated as the percentage of responses where all three digits were entered correctly relative to all presented trials. This was done separately for each typing task (STtype_aud, STtype_vis, MTtype_vis, MTtype_aud). Next, the reaction time of typing was calculated as the interval from stimulus onset until typing the first digit, again separately for each typing task. Reaction time outliers were eliminated with the ± 3.29 SD criterion^71^, separately for each participant and each typing task. Reaction time and accuracy were then averaged over the visual and the auditory typing tasks. To account for potential speed-accuracy trade-offs, we determined the inverted Balance Integration score (BIS), which puts equal weights on reaction time and accuracy [BIS^72^]. The pertinent formula is BIS = z (accuracy)–z (reaction time), where “z” refers to a z-transformation across all participants of a given task.

##### Crossing failures

The percentage of unsuccessful trials resulted from timeouts (i.e., participant did not complete street crossing within 80 s) or collisions (i.e., participant was hit by a car in the near or in the far lane). This outcome measure was chosen to operationalize successful and independent pedestrian mobility^56^.

##### Stay time

Time in s during which participants stood still at the curb while looking for a gap in traffic, averaged across all trials on a given task. This outcome measure was chosen to operationalize risk avoidance tendency^73^.

##### Crossing speed

Velocity in km/h while crossing the street, averaged across all trials on a given task. This outcome measure was chosen since walking speed is a common parameter in research on cognitive-motor multitasking performance^2^.

### Executive functions

A battery of eight computer-based tests, adapted from literature, was used to measure the executive functions inhibition (Simon test, Stroop test), updating (N-back test, Keep-track test), switching (Switch-semantic test, Switch-spatial test), and dual-tasking (Tracking-manual test, Tracking-verbal test), as described by^65^. Thus, each function was assessed with two tasks. Stimuli were presented on a 24″ monitor with 1920 × 1080 screen resolution or through loudspeakers. Each test took about 10 min and was preceded by standardized instructions and up to three practice trials of about 1 to 2 min each. Participants were instructed to respond as quickly and accurately as possible. Feedback was provided after the practice trials only.

#### Executive functions tests with E-Prime

Inhibition, updating, and switching was measured with E-Prime 2.0 (Psychology Software Tools, Pittsburgh, PA^74^). In each of those E-Prime based tests, stimuli were presented in six blocks with inter-block breaks of 5 s (20 s after the third block) and a maximum response time of 2000 ms. After a response was given or after the maximum response window, a central fixation cross was displayed for a variable response-stimulus interval between 2300 and 2700 ms for the Stroop test and between 800 and 1200 ms for all other trials. All stimuli were presented in black color on a white screen. Participants reacted by pressing the "X" key with the left index finger or the "M" key with the right index finger on a German keyboard.

##### *Simon test*^75^

Overall, 192 trials were presented. For the entire duration of each block, a fixation cross was presented in the screen center. Per block, 32 left- or rightward pointing arrows were displayed sequentially in a random order for 500 ms on the left or the right side of the fixation cross. For one-half of the stimuli, the direction and position of the arrow were congruent (e.g., rightward pointing arrow on the right side). In contrast, for the other half of the stimuli, the direction and position were incongruent (e.g., rightward pointing arrow on the left side). Participants were instructed to press the key “X” for arrows pointing to the left, and to press the key “M” for arrows pointing to the right, irrespective of the arrows position.

##### *Stroop test*^76^

Overall, 192 Trials were presented. Per block, 32 color-denoting words (i.e. yellow, red, blue, green) were displayed in yellow, red, blue or green font for 500 ms each, in random order. Font and meaning of words were compatible in congruent trials (e.g., the word “green” in green font) and were incompatible in incongruent trials (e.g., the word “green” in blue font). Two response options were displayed below each stimulus for 2000 ms in white font. One response option named the font color of the stimulus (correct response), and the other named one of the other font colors. The position of the correct response, on either the right or the left side, was randomized across trials. Participants were instructed to press the key “X” if the correct answer was presented on the left side, and the key “M” if the correct answer was given on the right side.

##### *Keep-track test*^77^

Overall, 90 trials were presented. Per block, 15 words from six categories (animals, colors, relatives, metals, countries, distances) were displayed in a random order in the center of the screen for 2000 ms each. Participants were asked to write down the last word from each category at the end of each block. The number of categories changed from block to block in the order 3,3,4,4,5,5.

##### *N-back test*^78^

A 4 × 4 grid was presented continuously on the screen. Overall, 114 trials were presented. Per block, a total of 19 black dots were presented sequentially in the center of the different cells. Participants were instructed to press the right “M” if the currently displayed dot was in the same position as the second-to-last dot, and otherwise to press the key “X”.

##### *Switch-semantic test*^79,80^

Per block, 17 words were presented sequentially in the center of the screen, for 1500 ms each. Word length (mono- or bisyllabic) and word meaning (animate or inanimate object) varied randomly from trial to trial. Participants were instructed to indicate whether the current word was mono- or bisyllabic (task A) or whether the word denoted an inanimate or an animate object (task B) by pressing either the key “X” for bisyllabic words or inanimate objects and the key “M” for monosyllabic words or animate objects. In each block, tasks A and B were presented in the order AABBAABB…, which allowed us to analyze switch trials (A preceded by B or B preceded by A) separately from repeat trials (A preceded by A or B preceded by B).

##### *Switch-spatial test*^79,80^

The test was similar to the Switch-semantic test, except that words were replaced by geometrical shapes and participants had to discriminate between quadratic and circular shapes (task A) or between small and big shapes (task B).

For all E-Prime presented tests, outlier elimination was done by first removing trials with reaction times < 80 ms or > 1300 ms and then using the ± 3.29 SD criterion^71^ per participant. Accuracy was then quantified as the percentage of correct responses across all presented stimuli and the mean reaction time of correct responses.

For the Keep-track test, only accuracy was taken as an outcome measure. For the other five tests, the outcome measure was the BIS, as for typing performance (see above). For the N-back test, BIS was calculated by quantifying accuracy and reaction time across target and non-target trials. For the Simon and the Stroop test, inhibition was quantified as BIS = z (accuracy incongruent trials–accuracy congruent trails)–z (reaction time incongruent trials–reaction time congruent trials). The BIS for the Switch-semantic and Switch-spatial test was calculated analogously: BIS = z (accuracy switch trials–accuracy repeat trials)–z (reaction time switch trials–reaction time repeat trials).

#### Dual-tasking tests

The two dual-tasking tests were performed with a customized program (for further information see^81,82^).

##### *Tracking-manual test*^81,82^

It consisted of a tracking task and a manual discrimination task. The tasks were presented separately (ST tracking task, ST manual discrimination task) and concurrently (dual-task (DT) tracking and manual discrimination). The tasks were presented in three blocks, each with three trials of the same task. Each of the nine trials had a duration of about 45 s. The order of blocks was mixed across participants.

In the ST tracking task, a small square red target moved along an unpredictable wave-shaped path from the left to the right edge of the screen. Participants were instructed to track the target as precisely as possible by controlling a small white crosshair cursor with a joystick grasped by their dominant hand.

In the ST manual discrimination task, ten high-pitched target sounds (1086 Hz) and 18 to 20 low-pitched distractor sounds (217 Hz or 652 Hz) were presented per trial in a random sequence. Sounds were presented through headphones for 75 ms each, with a variable interstimulus interval of 1000 to 1300 ms. Participants were instructed to respond to each target sound by pressing the “F12” key with their left index finger, and to refrain from responding to the distractor sounds.

In the DT tracking and manual discrimination task participants performed both single-tasks simultaneously. No instructions were provided regarding task prioritization.

##### *Tracking-verbal test*^81,82^

The test was exactly the same as the Tracking-manual test, except that a verbal discrimination task was used instead of the manual discrimination task. In this task, participants had to respond verbally with a “yes” to the target sounds.

For the discrimination task of the Tracking-verbal test and the Tracking-manual test, accuracy and reaction time were quantified similarly to the E-Prime presented tasks as percentage of correct responses across all presented stimuli and as mean reaction time of correct responses, separately for single-tasking and dual-tasking trials. For the tracking task, performance was assessed as root mean square error (RMSE) between the red target and the white cursor. Dual-tasking effects on the discrimination task were calculated for accuracy as [accuracy (dual-tasking)–accuracy (single-tasking)]/accuracy (single-tasking), and for reaction time as [reaction time (dual-tasking)–reaction time (single-tasking)]/reaction time (single-tasking). Dual-tasking effects on the tracking task were calculated as [RMSE (dual-tasking)–RMSE (single-tasking)]/RMSE (single-tasking). The outcome measure was calculated in two steps. First, the BIS was calculated for the dual-tasking effects on the discrimination task, similarly to the Stroop and the Simon test. The z-transformed dual-tasking effect score of the RMSE was then subtracted from the z-transformed BIS. A higher score represented better performance.

#### Outcomes

A composite score for executive function was quantified as the means of the z-transformed individual measures over the eight cognitive tests, as suggested in^65^. Test performances with mean overall ACC < 55% were classified as invalid tests, except for the Keep-Track test. Participants with less than seven valid tests were excluded from further analysis. This was the case for eight older adults.

### Motor coordinative fitness

#### Motor coordinative fitness tests

A battery of five tests, adapted from literature^25,83^ was used to measure three different domains of motor coordinative fitness: balance (One-legged stand test), movement speed (Hand tapping test, Feet tapping test, Timed-Up-and-Go test) and fine coordination (Purdue Pegboard test). Before each test, participants were familiarized with the procedure.

##### *One-legged stand test*^84^

Participants stood on one leg while looking straight ahead and slightly flexing the other leg for a duration of maximum 20 s (self-initiated). Overall, eight trials were performed, four trials with eyes open and then four trials with eyes closed (two trials each per leg). On each trial, time was stopped when participants put down their lifted foot, hopped, pushed the lifted leg against the standing leg, or opened their eyes during closed eyes trials. Performance was quantified as standing duration in s of the eyes closed balancing condition, averaged across two trials, the one with the highest standing duration of the right leg and the one with the highest standing duration of the left leg. Because of ceiling effects for the eyes open trials, only eyes closed trials were analyzed.

##### *Feet tapping test*^25^

The participants sat on a stationary, height adjustable chair without armrests. They were instructed to repeatedly move both feet as fast as possible back and forth across a line on the floor aligned with the participants’ mid-sagittal plane. A crossing was rated as correct if both heels touched the ground. Two trials were performed, each lasting 20 s. Performance was quantified as the number of crossings of the trial with the highest number of correctly performed crossings.

##### *Hand tapping test*^85^

Participants sat on the same chair as in the Feet tapping test, in front of a table. They placed the non-dominant hand on a line on the table that was aligned with their mid-sagittal plane and were asked to move the other hand as fast as possible back and forth over the resting hand, with the flat palm touching the table after each crossing. One trial was finished when the participants completed 25 back and forth movements. Two trials were performed. Performance was quantified as duration in s of the trial with the lowest duration.

##### *Timed-up-and-go test*^86^

Participants sat again on the chair. They were instructed to stand up, walk 1.80 m to a cone on the right side, walk around it, walk back, sit down, and then execute the same procedure to the left side. Three trials were performed. Performance was quantified as duration in s of the trial with the lowest duration.

##### *Purdue Pegboard test* (Model 32,020, Lafayette Instruments, Lafayette, IN, USA^87^)

Participants were instructed to place as many pegs as possible into two parallel columns of 25 small holes from top to bottom. During the first trial, they used their dominant hand and filled the column on the pertinent side. During the second trial, they used their non-dominant hand and the pertinent column. During the third and final trial, they used both hands to fill both columns simultaneously. Each trial lasted 30 s. Performance was quantified as average number of rows with correctly placed pegs over the three trials.

#### Outcomes

A composite score for motor coordinative fitness was calculated similarly to^83^, as the mean of the individual z-transformed outcome measures of the following three motor domains: movement speed (mean of the Feet tapping test, the Hand tapping test and the Timed-Up-and-Go test), balance (One-legged stand test) and fine motor coordination (Purdue Pegboard test). For the Hand tapping test and the Timed-Up-and-Go test, the inverted z-scores were used, such that higher values indicated better performance on all tests.

### Cardiovascular fitness

Spiroergometry (ZAN600 CPET, nSpire Health, Oberthulba, Germany) on a stationary bicycle (Lode Corival cpet, Groningen, the Netherlands) was conducted to assess cardiovascular fitness. Bike, hardware and protocol were similar to^88^ and^89^. The test followed first an alternating 30 W/80 W protocol that lasted about 10–15 min, which will be analyzed elsewhere. This was followed by a ramp protocol that lasted about 15 min and was similar to standard protocols reported in literature^27,83,90^. In the ramp protocol, the load increased from 30 W every minute by 10 W for females and 15 W for males. The ramp protocol was preceded by a resting-up period of 3 min and was followed by a cool-down period of 5 min (1 min 30 W, then 0 W). The following parameters were continuously monitored: (1) Breath-by-breath respiration (oxygen uptake in VO2), (2) carbon dioxide output in VCO2), (3) respiratory exchange ratio (RER = VCO2/VO2), (4) heart rate, (5) blood pressure (every two minutes), and (6) Electrocardiography (ECG, recorded with a ten-lead ECG fully digital stress system; Kiss, GE Healthcare, Munich, Germany). Perceived exertion was assessed every 2 min with the Borg-RPE-Scale (Borg 1982). As a precautionary measure, the protocol was terminated prematurely if risk factors occurred (i.e., RER > 1.10 or > 1.05 for at least 30 s, HR > about 220-age, blood pressure > 230/115 mmHg, dizziness, cardiac arrhythmia, or other abnormalities). An experienced sports scientist supervised testing.

#### Outcome

Cardiovascular fitness was determined as the average value of oxygen consumption (VO2) at the highest complete performance level (highest wattage level, held for min 3 min), expressed as VO2 peak ml/kg. For three participants, no cardiovascular fitness score could be determined due to technical problems; they were excluded from the present analyses. The outcome was the z-transformed individual cardiovascular fitness score.

### Procedure

**Figure 4:**
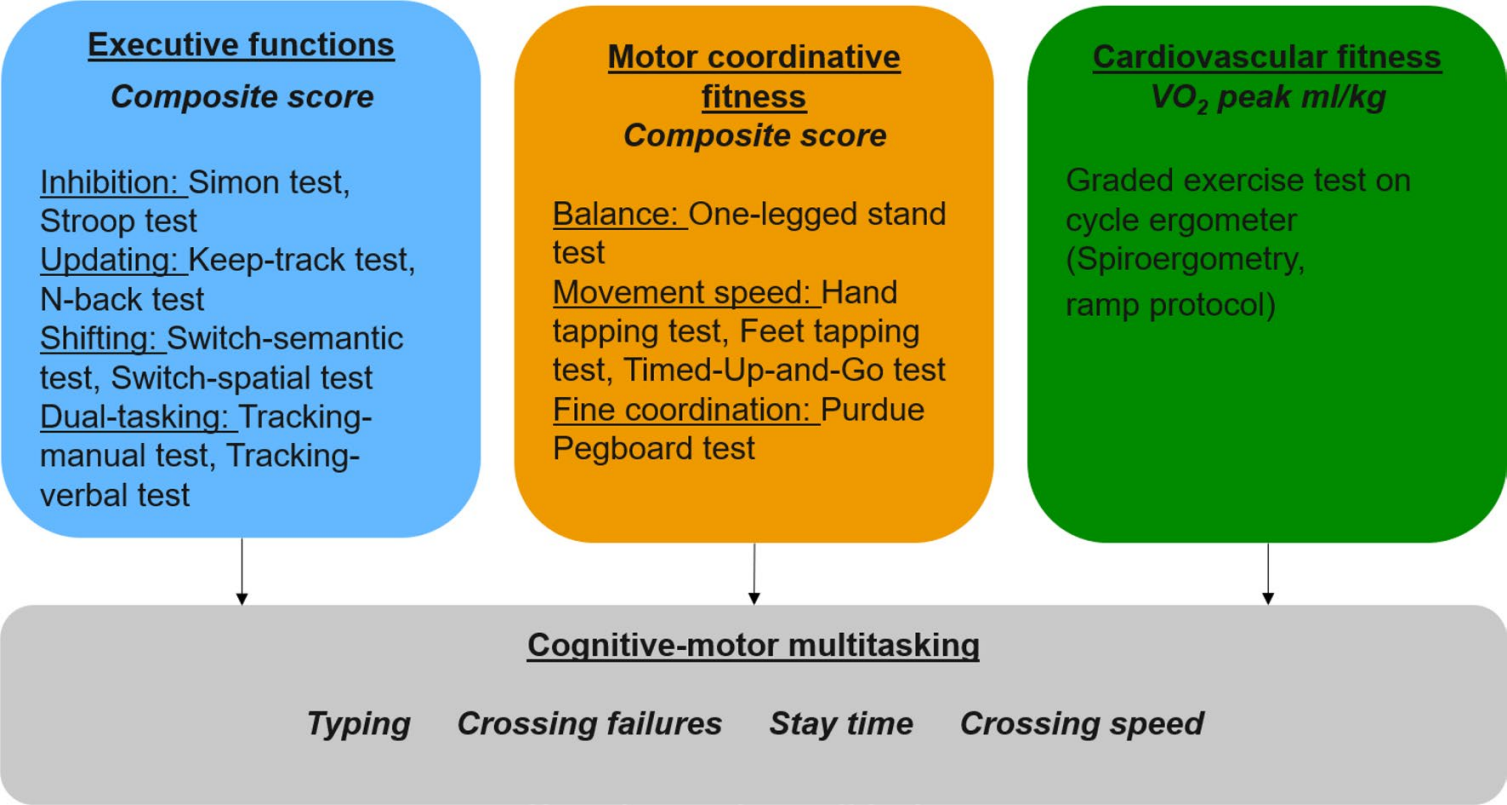
Overview of the assessment of executive functions, motor coordinative and cardiovascular fitness as well as the different components of cognitive-motor multitasking.

**Figure 5:**
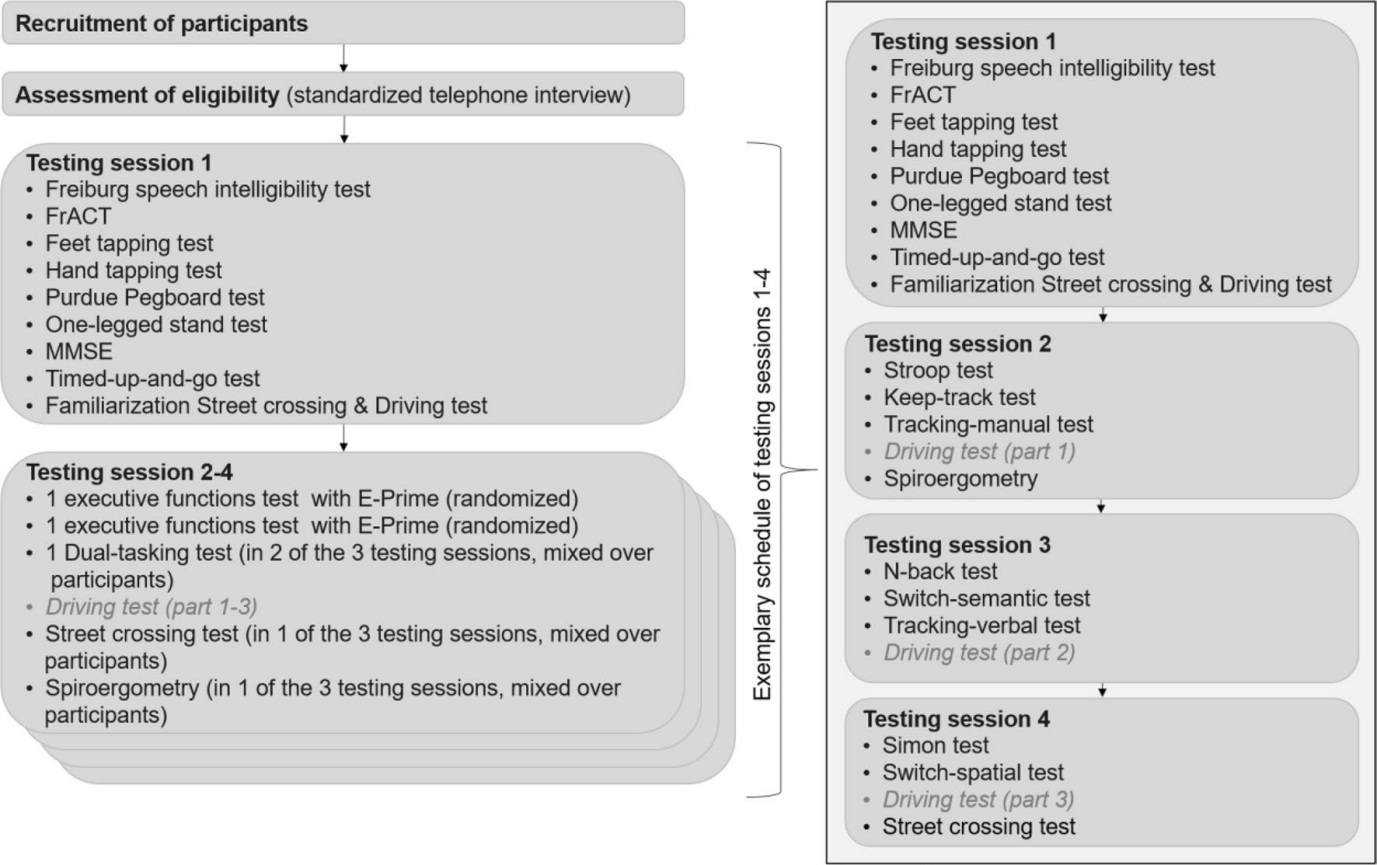
Study flow chart. Data of the driving test was not analyzed for this study and is therefore marked in grey and italic. FrACT = Freiburg Visual Acuity Test; MMSE = Mini Mental State Examination.

Before testing, all eligible participants gave written informed consent and filled out a questionnaire on personal and demographic information. All other data were collected in four sessions of approximately 2 h each, held one to seven days apart. The first session included screening tests, motor fitness tests, and familiarization with the treadmill and cognitive-motor multitasking test. During familiarization, they practiced starting, walking and stopping on the treadmill until the participant and the experimenter considered those actions to be smooth and consistent, which took about 15 to 20 min. Afterwards, they received one practice trial for each ST condition. MT conditions were not practiced. The remaining three test sessions included cognitive functioning tests, the cardiovascular fitness test and the cognitive-motor multitasking test. For an overview of the assessed outcome variables see Fig. 4 and for an overview of the study flow see Fig. 5. The cognitive-motor test took about 40 min in total. Participants were asked to avoid caffeine and alcohol intake 12 h and any vigorous physical activities 24 h before the cardiovascular fitness test.

### Statistical analysis plan

#### Sample size estimation

In order to answer the research question addressed here, data from a previous study were analyzed. We performed a sample size approximation before data analysis to approximately determine whether the available sample of *N* = 61 older adults is sufficient to find a medium effect for the predictors of interest. Sample size approximation was performed with GPower 3.0 using R^2^ increase for fixed model linear multiple regression (*α* = 0.05 and *ß* = 0.80). We assumed a medium effect size (*f*^2^ = 0.15) for each of the tested predictors, i.e., main effects of condition, executive functions, motor coordinative fitness, and cardiovascular fitness, and the interaction effects of condition with executive functions, motor coordinative fitness, and cardiovascular fitness respectively. In examining the lowest order interaction effect (i.e., two-way interactions) the number to tested predictors was set to ‘1’, and the number of total predictors was set to ‘8’, including three covariates, and all other main and interaction effects. The required sample size was estimated at *N* = 55. Since 61 participants were available for analysis, we were confident to perform the current analysis.

#### Linear mixed-effects model analysis

We applied linear mixed-effects model analysis to examine the effects of condition, executive functions, motor coordinative fitness, cardiovascular fitness, and their interactions on cognitive-motor multitasking (hypothesis I). Therefore, separate linear mixed-effects models were performed for each of the four dependent variables typing, crossing failures, stay time, and crossing speed. The model included the following fixed-effects terms: condition (multitask vs. single-task), executive functions, motor coordinative fitness, cardiovascular fitness, and the interaction of each parameter with condition. Covariates gender, age, and education were included as fixed-effects terms to control for those potential confounds. Random intercepts and slopes of participants for conditions were included as random-effects terms to control for non-independence in the data.

Before running the linear mixed-effects model analysis we tested multicollinearity and distributional assumptions. We used the variance inflation factor (VIF) to detect multicollinearity in our models. For all four models, the results revealed VIF scores smaller than five for all independent variables, indicating that multicollinearity was not a concern (see Supplementary Table S2). Normal distribution of the residuals was checked with the Shapiro–Wilk test. Typing was normally distributed (*W* = 0.99, *p* = 0.460). Distribution from crossing speed (*W* = 0.99, *p* = 0.044), stay time (*W* = 0.99, *p* < 0.001), and crossing failures (*W* = 0.99, *p*  <  0.001) departed significantly from normality. Since linear mixed-effects models are robust to violations of the distributional assumptions^91,92^, we applied them despite the non-normally distributed residuals.

Linear mixed-effects models were implemented using the “lmer” function of the “lme4” package^93^ using restricted maximum likelihood estimation. Further, we used Satterthwaite's method to estimate degrees of freedom and significance. Significance level was set at α = 0.05. In case of significant interaction effects, post-hoc linear mixed-effects models were calculated separately for single- and multitask conditions including executive functions, motor coordinative fitness, and cardiovascular fitness as fixed-effects terms as well as the covariates gender, age, and education.

#### Commonality analysis

To examine the relative and interrelated contribution of executive functions, motor coordinative fitness, and cardiovascular fitness for the different cognitive-motor multitasking outcomes (hypothesis II), a commonality analysis was performed^58,59^. With that, we determined the extent to which the independent variables executive functions and motor coordinative and cardiovascular fitness alone and in combinations explained variance of the dependent variables typing, crossing failures, stay time, and crossing speed. Commonality analysis was performed for each of the four dependent variables, separately for single- and multitask conditions, with the ‘yhat’ package (Kim et al. 2021). The unique and the shared variance explained by executive functions, motor coordinative fitness, and cardiovascular fitness was calculated. Gender, age and education were included to account for potentially confounding variables. Correlations between dependent and independent variables were conducted and are presented in the appendix (see Supplementary Table S1).

### Ethical approval and informed consent

The studies involving human participants were reviewed and approved by the ethics committee of the German Sport University, Cologne, Germany (Nr.: 27/2015) and the ethics committee of the Chemnitz University of Technology, Germany (Nr.: V-280-17-CVR-Multitasking-29062018). The participants provided their written informed consent to participate in this study.

## Supplementary Information

Supplementary Tables.

## Data Availability

The raw data supporting the conclusions of this article will be made available by the authors, without undue reservation. Requests to access the datasets should be directed to CV-R (claudia.voelcker-rehage@uni-muenster.de).
