# Supplementary material for: The association of executive functions and physical fitness with cognitive-motor multitasking in a street crossing scenario
Source: Sci Rep. 2023 Jan 13;13:697. doi: 10.1038/s41598-022-26438-x (PMC9839686; doi:10.1038/s41598-022-26438-x)
Supplement: Supplementary file 1 — Supplementary Tables. [file 41598_2022_26438_MOESM1_ESM.docx]

**Supplementary Table S1.** Correlations between dependent and independent variables.

| Variables | 2 | 3 | 4a | 4b | 5a | 5b | 6a | 6b | 7a | 7b |
| --- | --- | --- | --- | --- | --- | --- | --- | --- | --- | --- |
| 1. EF | -0.02 | 0.09 | 0.18* | 0.40** | -0.06 | -0.02 | 0.02 | 0.09 | -0.35** | -0.30** |
| 2. MF |  | 0.07 | 0.06 | 0.20* | -0.29** | -0.31** | 0.20* | 0.24** | -0.05 | -0.11 |
| 3. CF |  |  | 0.34** | 0.10 | 0.05 | 0.01 | 0.10 | 0.08 | -0.35** | -0.05 |
| 4a. ST typing |  |  |  | 0.51** | 0.15* | 0.06 | 0.12 | 0.16* | -0.30 | -0.28** |
| 4b. MT typing |  |  |  |  | -0.11 | -0.11 | 0.21* | 0.21* | -0.22 | -0.14* |
| 5a. ST stay time |  |  |  |  |  | 0.77** | -0.25** | -0.12 | -0.14 | -0.28** |
| 5b. MT stay time |  |  |  |  |  |  | -0.04 | 0.02 | -0.06 | -0.41** |
| 6a. ST crossing speed |  |  |  |  |  |  |  | 0.91** | -0.29** | -0.32** |
| 6b. MT crossing speed |  |  |  |  |  |  |  |  | -0.40** | -0.52** |
| 7a. ST crossing failures |  |  |  |  |  |  |  |  |  | 0.52** |
| 7b. MT crossing failures |  |  |  |  |  |  |  |  |  |  |

* *p* < .05, ** *p* < .001.

**Supplementary Table S2.** Variance inflation factors (VIF) of the dependent variables in the linear mixed-effects models for typing, crossing failures, stay time, and crossing speed.

| **Dependent variable** | **Typing [BIS]** | **Crossing failures [%]** | **Stay time [s]** | **Crossing speed [km/h]** |
| --- | --- | --- | --- | --- |
| Gender | 1.95 | 1.95 | 1.95 | 1.95 |
| Age | 1.10 | 1.10 | 1.10 | 1.10 |
| Education | 1.12 | 1.12 | 1.12 | 1.12 |
| Cond | 1.00 | 1.00 | 1.00 | 1.00 |
| EF | 1.44 | 1.73 | 1.39 | 1.18 |
| MF | 1.48 | 1.76 | 1.42 | 1.21 |
| CF | 2.25 | 2.54 | 2.20 | 1.99 |
| Cond * EF | 1.39 | 1.69 | 1.34 | 1.13 |
| Cond * MF | 1.39 | 1.68 | 1.34 | 1.13 |
| Cond * CF | 1.40 | 1.69 | 1.35 | 1.14 |
